# Comparative study of eco-friendly wire mesh configurations to enhance sustainability in reinforced concrete structures

**DOI:** 10.1038/s41598-024-59050-2

**Published:** 2024-04-17

**Authors:** Misgina Mebrahtom, Yewuhalashet Fissha, Mujahid Ali, Angesom Gebretsadik, Yemane Kide, Zaid Nguse, Zemicael Gebrehiwot, Erick Saavedra Flores, Siva Avudaiappan, Hajime Ikeda

**Affiliations:** 1 Department of Civil Engineering, Ethiopia Institute of Technology, Mekelle/EIT-M, Tigray, Ethiopia; 2 Department of Geosciences, Geotechnology and Materials Engineering for Resources, Graduate School of International Resource Sciences, Akita University, Akita, 010-8502 Japan; 3 Department of Mining Engineering, Aksum University, 7080 Aksum, Tigray, Ethiopia; 4 Department of Transport Systems, Traffic Engineering and Logistics, Faculty of Transport and Aviation Engineering, Silesian University of Technology, Krasińskiego 8 Street, Katowice, Poland; 5 Department of Hydraulic Engineering, Aksum University, 7080 Aksum, Tigray, Ethiopia; 6 Departamento de Ingeniería en Obras Civiles, Universidad de Santiago de Chile, Av. Ecuador 3659, Estación Central, Santiago, Chile; 7 Departamento de Ciencias de la Construcción, Facultad de Ciencias de la Construcción y Ordenamiento Territorial, Universidad Tecnológica Metropolitana, Santiago, Chile

**Keywords:** Strength properties, Eco-friendly, Welded square wire mesh, Lightweight aggregate concrete, Sustainability, Engineering, Materials science

## Abstract

Recent and past studies mainly focus on reducing the dead weight of structure; therefore, they considered lightweight aggregate concrete (LWAC) which reduces the dead weight but also affects the strength parameters. Therefore, the current study aims to use varied steel wire meshes to investigate the effects of LWAC on mechanical properties. Three types of steel wire mesh are used such as hexagonal (chicken), welded square, and expanded metal mesh, in various layers and orientations in LWAC. Numerous mechanical characteristics were examined, including energy absorption (EA), compressive strength (CS), and flexural strength (FS). A total of ninety prisms and thirty-three cubes were made. For the FS test, forty-five 100 × 100 × 500 mm prism samples were poured, thirty-three 150 × 150 × 150 mm cube samples were made, and forty-five 400 × 300 × 75 mm EA specimens were costed for fourteen days of curing. The experimental findings demonstrate that the FS was enhanced by adding additional forces that spread the forces over the section. One layer of chicken, welded, and expanded metal mesh enhances the FS by 52.96%, 23.76%, and 22.2%, respectively. In comparison to the remaining layers, the FS in a single-layer hexagonal wire mesh has the maximum strength, 29.49 MPa. The hexagonal wire mesh with a single layer had the greatest CS, measuring 36.56 MPa. When all three types of meshes are combined, the CS does not vary in this way and is estimated to be 29.79 MPa. In the combination of three layers, the chicken and expanded wire mesh had the most energy recorded prior to final failure, which was 1425.6 and 1108.7 J, whereas it was found the highest 752.3 J for welded square wire mesh. The energy absorption for the first layer with hexagonal wire mesh increased by 82.81% prior to the crack and by 88.34% prior to the ultimate failure. Overall, it was determined and suggested that hexagonal wire mesh works better than expanded and welded wire meshes.

## Introduction

Structure stability and enhancement of strength properties are the main goals of engineers. However, researchers mostly considered green and sustainable construction while enhancing the strength properties; therefore, they try to limit the use of natural resources and widely used waste materials^1^. Recently, several innovations have been introduced in the construction industry globally in which concrete-related innovations took the lion’s share of the current period. However, with those innovations, still have several deficiencies need to be resolved. Concrete consumes a substantial amount of natural resources like cement, coarse and fine aggregate which is tread to the environment^2,3^. According to Alexander and Sakthivel^4,5^, to reduce the use of natural resource consumption and achieve sustainability in buildings, we should utilize abundantly available waste material as a natural resource replacement. It is vitally necessary to use creative, green, lightweight, and affordable materials, particularly those that can serve the intended purposes, of preserving the world's natural environment and guaranteeing the sustainability of natural resources^6–9^.

Apart from the natural resources, plain concrete has several challenges such as low tensile strength, ductility, high porosity especially in severe service conditions, and resistance to crack propagation^10^. Recent and past studies took several approaches to improve concrete properties, resulting in quite different materials^11–15^. Serviceability criteria such as excessive crack width and deflection impair the appearance of the structure, weakening the member due to corrosion of steel and damaging non-structural members become more critical than the strength consideration^16^. Due to the technological development, recent studies focusing on the utilization of modern techniques such as artificial intelligence and modern techniques in concrete industry for the prediction of strength parameters by utilizing several wastes and additives^2,17–19^. A repair good improves the function and performance of structures, restores and increases their strength, provides water tightness, and prevents aggressive environment to steel surface durability^20^.

Despite the disadvantages of traditional concrete, there are some benefits of plain concrete. It's critical to keep a balanced viewpoint while debating the benefits and drawbacks of conventional materials such as plain concrete. Conventional concrete is the material of choice for many different construction applications because of its well-known strength and durability. One of its main advantages is that it can endure large loads and challenging weather. Conventional concrete is highly versatile, fire resistant, and has a low maintenance cost once properly installed. Corrosion of reinforced cement concrete structures takes place in the main reinforcement in the slab, beam, and stirrups, where cover is not provided well. To overcome such problems in the construction industry, Ferrocement, among other materials, has emerged. There are certain similarities and differences between reinforced concrete and ferrocement materials, indicating that ferrocement requires a separate investigation to determine its structural performance^21^.

The material Ferrocement is defined as a material type of thin wall-reinforced concrete structure commonly constructed of hydraulic cement mortar reinforced by small-sized wire mesh as per the committee of American concrete institute^22^. It has a number of special qualities, benefits, and challenges. Ferrocement is the first and oldest form of reinforced concrete which was used in France and Italy for the construction of boats dating back two centuries^23,24^, whereas its use in building construction began in the middle of the twentieth century in Italy. Ferrocement laminates are used to improve the overall performance of constructions including composite bridge decks, beams, and bearing walls^5,25,26^. Although its application in a large number of fields has rapidly increased all over the world, the state-of-the-art of Ferro cement is still in its infancy, as its long-term performance is still unknown.

Ferrocement is generally cast by encapsulating the steel mesh with a properly designed cement mortar (made of cement and natural sand, with no coarse aggregate like gravel) in smaller thicknesses (ranging from 10 to 25 mm) which usually gives high strength due to the availability of reinforced wire mesh^27^. The construction of ferrocement structures requires specialized skills and expertise, particularly in terms of forming and applying the cement mortar and reinforcement. In Ferrcement, the cement matrix does not crack since cracking forces are taken over by wire mesh reinforcement immediately below the surface. However, corrosion is one of the primary concerns that must be addressed in order to improve the long-term service life of ferrocement composites^28^. After this, it is not surprising that the brittle nature of concrete is an inherent property of the material and one that is overcome using reinforcing materials. As stated by Prathima and Jaishankar^29^ due to the close spacing interlocks of the steel meshes the reinforced concrete member provides good ductility and bearing capacity. Recent and past studies concluded that by providing an additional layer of wire mesh improves FS, EA, and cracking behaviour. A substantial number of longitudinal mesh wires allow for a bond transfer between the cement matrix and the reinforcing mesh, which improves toughness and impact resistance while also demonstrating good crack control^27^.

Ferrocement has several advantages, including strength, durability, and versatility, but it also comes with challenges such as the need for skilled labor, quality control, and potential brittleness. Recent and past studies used cement mortar construction materials to study the strength parameters such as Lesovik et al.^30^, studies the mortar for 3D printing by mineral modifiers to improve the performance, while Barreto et al.^12^, used the clay ceramic as a pozzolan constituent in cement for structural concrete. Moreover, Alani et al.^31^, studied demolishing waste and used in concrete to completely replace the cement and develop cement-free binders. Murali et al.^32^, studied the crumb rubber as a aggregate replacement from 5 to 30% with 5% increment and concluded that it enhance the initial crack and failure impact energy absorption capacity. Recently, researchers studied the confinement methods at different angles and spacing to enhance the strength properties of concrete^33^. For instance, Ali et al.^34^, studies the confinement behaviour low strength concrete under axial compression using experimental and analytical approaches.

Moreover, latest studies used modern techniques such as artificial intelligence and machine learning techniques to study mechanical properties of concrete incorporating different wastes to enhance the model stability and efficiency. For instance, Nafees et al.^35^, studied the mechanical properties of silica fume-based green concrete using ML techniques. Asghar et al.^8^, compiled a review on the structural and mechanical performance of geopolymer concrete to promote green and sustainable construction. Moreover, LWAC is widely used in the construction industry by using several conventional and analytical tools. Room et al.^36^, studies the LWAC incorporating textile washing stone, whereas Ali et al.^37^, applied central composite design application using response surface methodology to study the strength properties of LWAC incorporating pumice stone.

Several studies have been conducted using confinement behaviour in beams and columns at different angles and spacing to enhance the strength properties. Past researchers mostly focus on the utilization of waste materials as a natural aggregate replacement to reduce the use of natural resources, enhance strength parameters, and promote a green and sustainable environment. The latest studies in the concrete industry focus on the utilization of modern techniques such as AI and ML for the prediction of strength properties and claim that modern techniques outperformed conventional techniques. However, according to the author's view and past studies, there has been a limited study on ferrocement by using different layers of several wire meshes in LWA concrete to investigate the mechanical properties and energy absorption. Therefore, the current study aims to use varied steel wire meshes such as hexagonal (chicken), welded square, and expanded metal mesh in various layers and orientations to investigate the effects of LWAC on mechanical properties.

## Materials and methods

The materials used in the research process were cement, fine aggregate and coarse aggregate, three types of gabions (welded wire mesh, hexagonal wire mesh and expanded metal wire mesh) reinforcement bar, super plasticizer admixture and potable water. The physical and chemical composition properties of these materials have been duly investigated.

The chemical reaction associated with the decarbonation of limestone at high temperature, which produces cement, releases a substantial amount of carbon dioxide^38^. Ordinary portland cement (OPC) used in this study which satisfies the standard of the building codes of Ethiopia. The specific gravity properties of OPC were 3.15, Standard consistency was 34% and initial setting time was 40 min. The consistency and setting time test was conducted based on the ASTM-C191-19^39^. The curing days greatly affect the concrete compressive strength. On 28 days of curing, the concrete gains the highest strength. The current study chose 14 curing days due to the superplasticizer admixture with 0.2% in concrete which enhances earlier concrete strength. An admixture known as superplasticizer with a proportion of 0.2% was used in order to facilitate the strength-gaining period of the concrete. Potable water readily available in the local area was used which satisfies the drinking standard of Ethiopia.

River sand readily available which satisfies the requirement to be used in concrete casting was used. The sieve analysis of fine aggregate is conducted in the laboratory to study the physical properties as depicted in Table 1. Lightweight sand provides the same tensile strength as natural sand but lowers the compressive strength of Ferro-cement specimens^40^.

**Table 1:** Sieve analysis of fine aggregate.

Sieve size in mm	Weight of sieve (gm)	Weight of sand + retained sand(gm)	Mass retained	Percent retained (%)	Cumulative percent retained (%)	Percentage passing
4.75	449	494	45	2.25	2.25	97.75
2.36	442	507	110	5.5	7.75	92.25
1.18	493	660	167	8.35	16.1	83.9
0.6	397	1282	885	44.25	60.35	38.65
0.3	362	978	616	30.8	91.15	8.85
0.15	341	498	157	7.85	99	1
Pan	363	381	19.9	1	100	0

**Figure 1:**
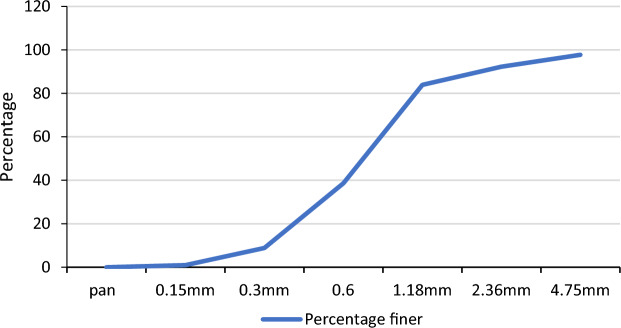
Gradation of sand.

The sand used in the research was tested for its basic properties. The laboratory tests made fine aggregate are sieve analysis, specific gravity, and silt content tests. The majority of the sand used was passed through a 4.75 mm sieve. The sieve analysis test was performed based on ASTM -C136/C136M-19^41^. The gradation test result shows that the sand is well-graded sand with a fineness modulus of 2.25 as shown in Fig. 1.

Since the presence of more silt or organic matter made concrete or mortar decrease the bond between the materials to be bound together and hence the strength of the mixture. The finer particles do not only decrease the strength but also the quality of mixture produced resulting in fast deterioration. Therefor it is necessary that one make a test on the silt content and checking against permissible limits. According to the Ethiopian Building Code Standard, if the silt content of the sand is more than 6% it shall not be used for construction. But the result (2.45% < 6%) complies with the standard and we used the sand material as shown in Table 2.

**Table 2:** Silt content of fine aggregate.

Sample number	Amount of silt deposit above the sand (A)	Amount of clean sand (B)	Silt content = (A/B) * 100%
1	1 mm	46.5	2.15%
2	1.5	48	3.13%
3	1.2	48.5	2.06%
Average silt content			2.45%

The main objective of the laboratory test is to determine the specific gravity and the water absorption capacity of fine aggregate. The test has been made according to ASTM-C-128-97 manual. Though the aggregates and sand we used were from a construction site on the main campus it has been found that duly studying the behavior of the materials is an important stage since it affects the final output of the concrete cast by the materials. Table 3 depicted the outcomes of specific gravity and water absorption of fine aggregate.

**Table 3:** Specific gravity and water absorption of the sand according to ASTM-C-128-97.

No	Trial number	1	2	3	Average
1	Weight of oven dry specimen in air, in gm (A)	458	452	464	458
2	Weight of pycnometer filled with water, gm (B)	657	657	657	657
3	Weight of with specimen and water to the calibration mark, gm (C)	934	940	949	941
4	Weight of saturated surface dry specimen, gm (S)	500	500	500	500
5	Bulk specific gravity = A/(B + S − C)	2.05	2.083	2.23	2.12
6	Bulk specific gravity (SSD) = S/((B + S − C)	2.24	2.30	2.40	2.31
7	Apparent specific gravity = A/(B + A − C)	2.53	2.67	2.70	2.63
8	Absorption capacity = [(S − A)/A] * 100	9.1	10.62	7.76	9.1

**Figure 2:**
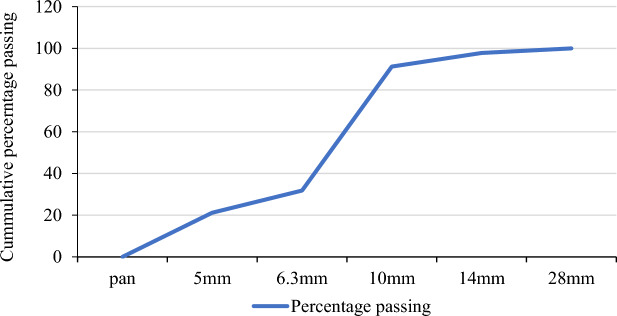
Gradation chart of coarse aggregate.

The aggregate that has used in the research had been examined for the fulfilment of ASTM-C-136-01 sieve analysis results and ASTM-C-127-88 standard test results for specific gravity determination^42^. Since the main intention of this research is to add aggregate for Ferro cement structures examining the gradation of the aggregate to be used is important. As a result, the sieve analysis results of the aggregate are depicted in Table 4. The aggregate has a maximum size of 14mm and most of the aggregate is retained in the 5mm sieve as shown in Fig. 2. Since some codes recommend not using materials that are finer than 5 mm as aggregate a sieving process has been made before casting the concrete.

**Table 4:** Sieve analysis result of the aggregate ASTM-C-136-01.

Sieve size in mm	Weight of sieve (gm)	Weight of sand + retained sand(gm)	Mass retained (gm)	Percent retained (%)	Cumulative percent retained (%)	Percentage passing
28	0.761	0.761	0	0	0	100
14	1.357	1.468	111	2.22	2.22	97.78
10	1.328	1.612	324	6.48	8.70	91.30
6.3	0.728	3.7	2972	59.44	68.14	31.86
5	0.712	1.247	535	10.7	78.84	21.16
Pan	0.759	1.854	1058	21.16	100	0

It is vital to determine the dry density in order to make the mix design calculations as well as to decide the compressive strength of the final cast concrete and as Ferro cement is a lightweight structure, we wanted to improve the material by lightweight aggregate in order to make it light as much as possible. The dry density of coarse aggregate is shown in Table 5. The specific gravity may be expressed as bulk specific gravity, bulk specific gravity [saturated surface dry (SSD)] or apparent specific gravity as shown in Table 6. Those parameters are used to determine the volume requirements and in determining the mix ratio calculations based on mass.

**Table 5:** Dry density of the coarse aggregate.

Trial no	Wight of the mold in kg (A)	Wight of the mold + filled aggregate in kg (B)	Wight of the retained aggregate	Volume of the mold (m^3^)	Density in (kg/m^3^)
1	2.968	4.845	1.877	3.21536*103	583.76
2	2.968	4.907	1.939	3.21536*103	603.04
3	2.968	4.898	1.930	3.21536*103	600.24
Average density					595.68

**Table 6:** Specific gravity and water absorption coarse aggregate ASTM-C-127–88.

Trial no	Wt. of oven dry sample (gm)	Wt. of SSD sample (B)	Wt. of SSD in water (C) (gm)	Bulk specific gravity A/(B − C)	Bulk specific gravity B/(B − C)	Apparent specific gravity A/(A − C)	Water absorption capacity in %
1	989	1000	548.3	2.19	2.21	2.24	1.1
2	990	1000	554.1	2.22	2.24	2.27	1
3	989	1000	555.5	2.22	2.25	2.28	1.1
Average values				2.21	2.23	2.26	1.07

Gabion (steel wire) meshes are thin steel wires has served in many fields of real-life applications though in different orientations and different mechanisms of applications. Most of the applications of gabion include that uses as a fence, uses in soil and water conservation works to prevent excessive erosion, and in some developed societies as building decoration works as well. The materials have very flexible behavior to be used in making different forms of ornamental works like different shapes in places where people used to recreate.

Different types of meshes are available almost in every country in the world. Two important reinforcing parameters are commonly used in characterizing Ferro cement and are defined as the volume fraction of reinforcement; it is the total volume of reinforcement per unit volume of Ferro cement. The specific surface of the reinforcement is the total bonded area of reinforcement per unit volume of the composite. The principal types of wire mesh currently being used in this research are hexagonal (chicken) wire mesh welded square wire mesh and expanded metal mesh among the available steel wire meshes. The addition of wire mesh layers as reinforcement improves flexural strength, cracking behavior, and energy absorption capability greatly.

**Figure 3:**
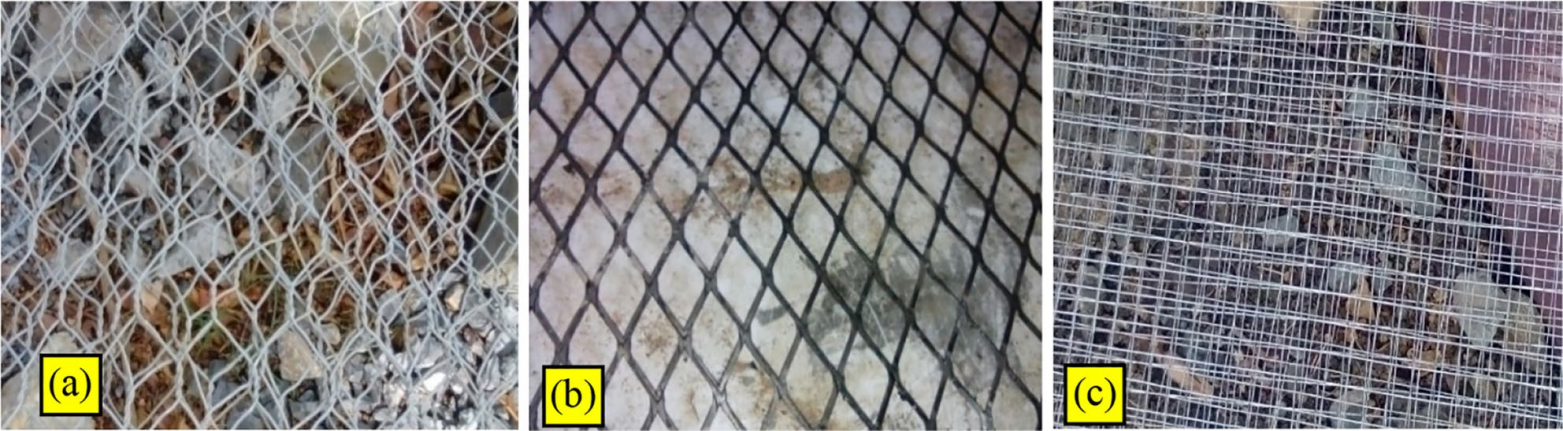
Different types of mesh (**a**) Hexagonal (chicken) wire mesh, (**b**) Welded square wire mesh (**c**) Expanded metal mesh.

Hexagonal or chicken wire mesh is readily available in most countries, and it is known to be the cheapest and easiest to handle. The mesh is fabricated from cold drawn wire which is generally woven into hexagonal patterns. Special patterns may include hexagonal mesh with longitudinal wires. The chicken wire mesh used in this research has a thickness of 2.2mm and opening spacing of 35mm as shown in Fig. 3a. The yield strength of the steel wire mesh is considered as 450Mpa as taken from the manufacturer’s specifications.

In welded square wire mesh, a grid pattern is formed by welding the perpendicular intersecting wires at their intersection. This mesh may have the advantage of easy molding into the required shape; it has the disadvantage of the possibility of weak spots at the intersection of wires resulting from inadequate welding during the manufacture of the mesh. Welded square wire mesh with a thickness of 0.7mm was used during the research as shown in Fig. 3b.

Expanded metal mesh is formed by cutting a thin sheet of expanded metal to produce diamond shape openings. It is not as strong as woven mesh, but on cost to strength ratio, expanded metal has the advantage. This type of mesh reinforcement provides good impact resistance and crack control, but they are difficult to use in construction involving sharps curves as shown in Fig. 3c.

As recommended by^43^ that the design strength for the mesh reinforcement shall be based on the yield strength ***f***_***y***_ of the reinforcement but shall not exceed 690 N/mm^2^. Design yield strengths of various mesh reinforcements are shown in the Table 7 as per the data from the material manufacturers and recommendations of Sharma studies. These shall be used for design only when test data are not available. In the research, we used this data for qualitative comparisons of the results got from the laboratory tests.

**Table 7:** Minimum values of yield strength and effective modulus for steel meshes and bars recommended for design.

		Welded square wire mesh	Hexagonal mesh	Expanded metal mesh	Longitudinal bars
Yield strength	fy N/mm^2^	310	450	310	300
Effective modulus	(Er) Long. (N/mm^2^)	104,000	200,000	138,000	200,000
	(Er) Trans. (N/mm^2^)	69,000	200,000	69,000	–

### Mix design

The chemical composition of the cement, the nature of the fine aggregate, coarse aggregate, and the water-cement ratio are the major parameters governing the properties of the concrete. The concrete matrix is designed for its appropriate strength and maximum denseness and impermeability, with sufficient workability to minimize voids and to avoid map cracking. Cement mortar used in ferro concrete acts as a good insulator and the reinforcing wire mesh can reduce surface upheaval better than plain concrete^44^. Precautions are necessary to maintain the small cover and in the selection of aggregates, mixing, placing, and curing. Mortar recommended for Ferro cement shall comprise particles or aggregates of limited size. The mortar matrix usually comprises more than 95 percent of the Ferro cement volume and has a great influence on the behavior of the final product. The cement mortar should be mixed with a proper sand-cement ratio (ranging from 1.5 to 2.5 by weight) and water-cement ratio (between 0.35–0.45 by weight) in order to achieve sufficient plasticity and facilitate easy casting. Many defects are possible due to a lack of complete infiltration and consolidation.

**Figure 4:**
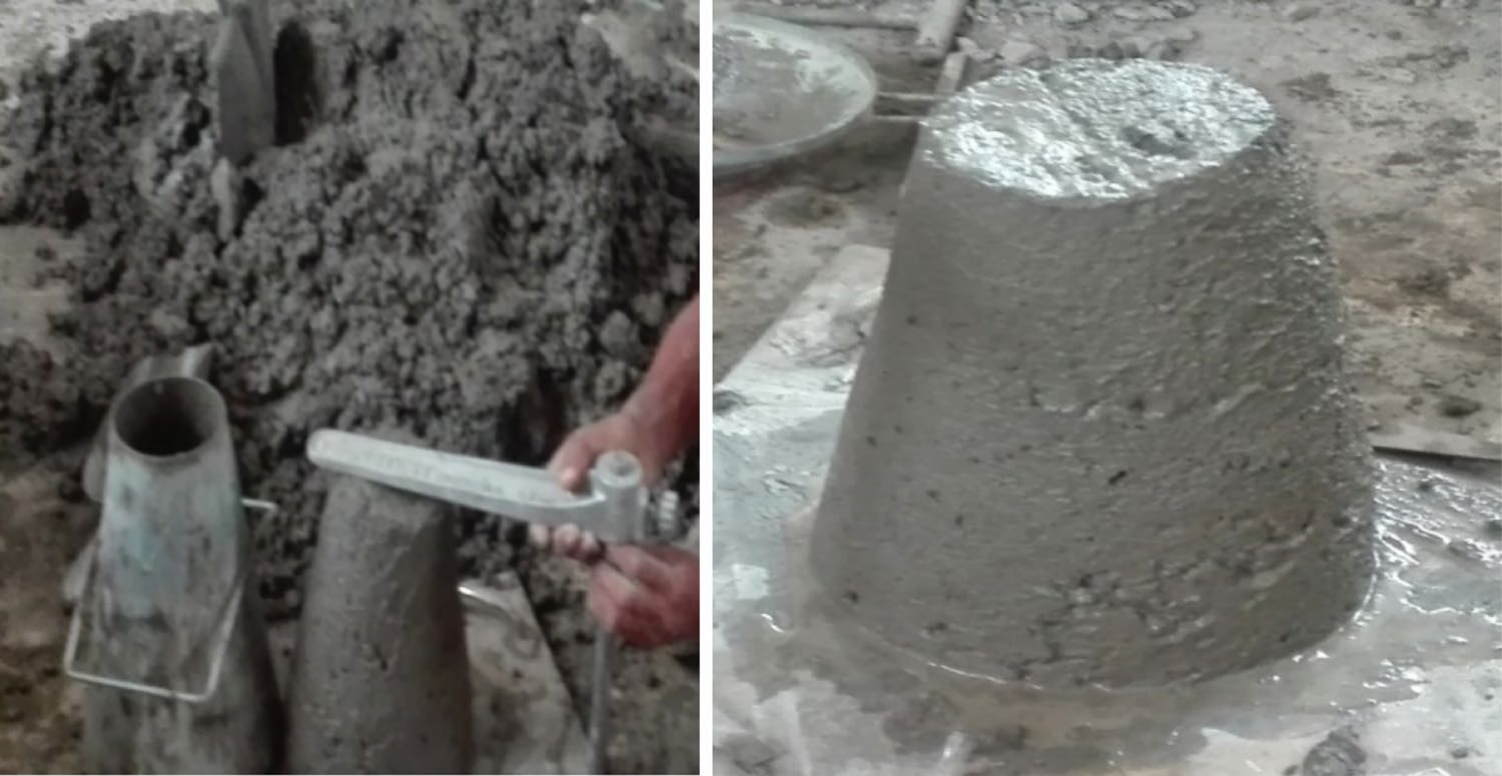
Workability test of concrete.

To avoid caking, the ingredients for mixing concrete, including the water, should be precisely batched by weight before poured into the mixer^45^. The sand-cement ratio should be calibrated to generate a fluid mix for the first infiltration of the armature, followed by a stiffer, more highly sanded mix at the finish, whereas the w/c should be as low as feasible^46^. As recommended that the CS should be at least 35 MPa for 28 days of curing with cube dimension of 150 × 150 mm^47^. According to the recommendations for ferroconcrete, we have used a water-cement ratio of 0.4 and a material proportion of 1:1.5:2 (cement, sand, and coarse aggregate respectively to cast a concrete of grade more than 35 Mpa. Since the concrete material is expected to possess good strength in a small thickness, the result is considered as ferro cement material improved by the addition of coarse aggregate of maximum size 14 mm taking in to account the opening of the gabion. Normally the slump of fresh concrete we cast was a true slump with 37mm as shown in Fig. 4. Admixtures or additives have been added to improve the performance and workability of the concrete.

Due to the lack of readily available standard molds in the laboratory temporary molds were prepared for the flexural specimens and for the energy absorption test specimens. The molds were prepared in such a way that the dimension of the inner mold satisfies the requirement of the standard molds. Two types of molds were prepared with inner dimensions of L:W:D (500:100:100) in mm for flexural mold and the second type slabs for energy absorption test with inner dimensions L:W:D is 400mm:300mm:75mm, by considering the two-way aspect ratio and the deep beam effect which shall be greater than 4 to avoid deep beam effect.

### Experimental plans

With a large number of samples, the study would have greater statistical power, meaning it would be better able to detect true differences or relationships between variables. This is important for drawing accurate conclusions and making reliable predictions. While increasing the sample size may require additional resources and time, the benefits in terms of the study's validity and reliability often outweigh these costs. Researchers in concrete research should aim to optimize their sample sizes to ensure that their findings are robust and applicable to real-world scenarios. Therefore, considering the aspect of time and money, the authors chose ninety prisms and thirty-three cubes for their experimental design which is sufficient enough to draw accurate conclusions and make reliable predictions.

#### Flexural tests

**Figure 5:**
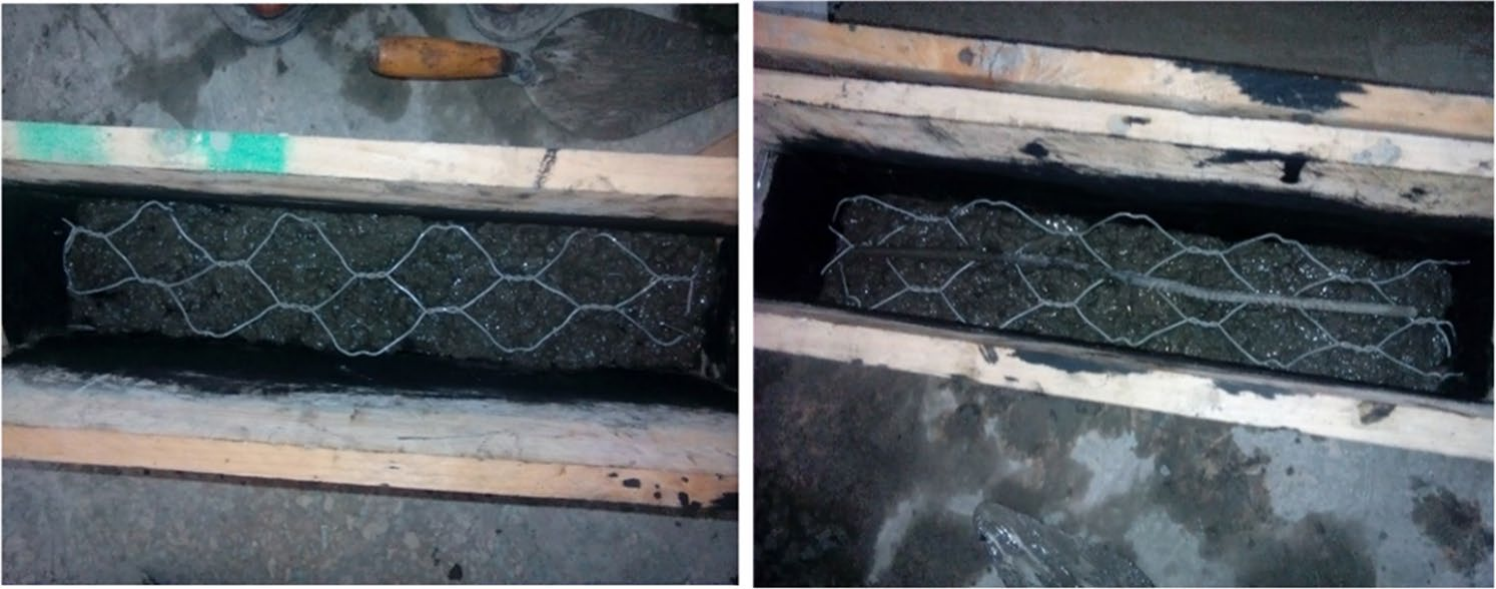
Casting of ferro concrete beam model using hexagonal (chicken) wire mesh.

**Figure 6:**
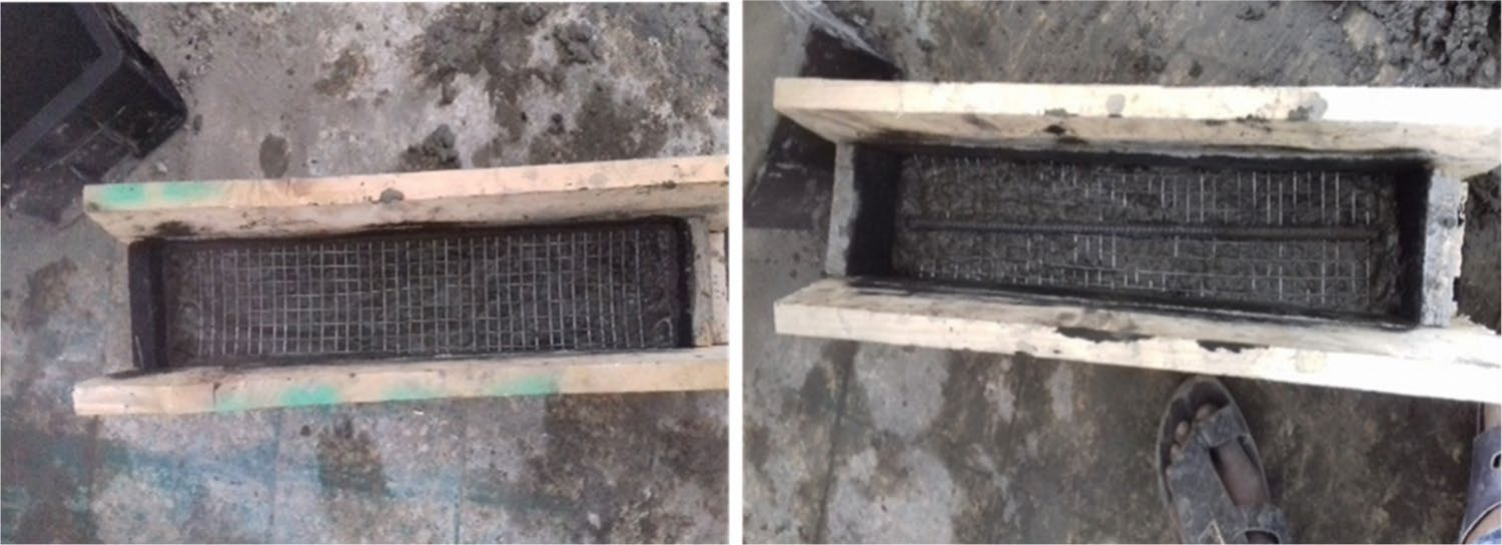
Casting of ferro concrete beam model using welded square wire mesh.

**Figure 7:**
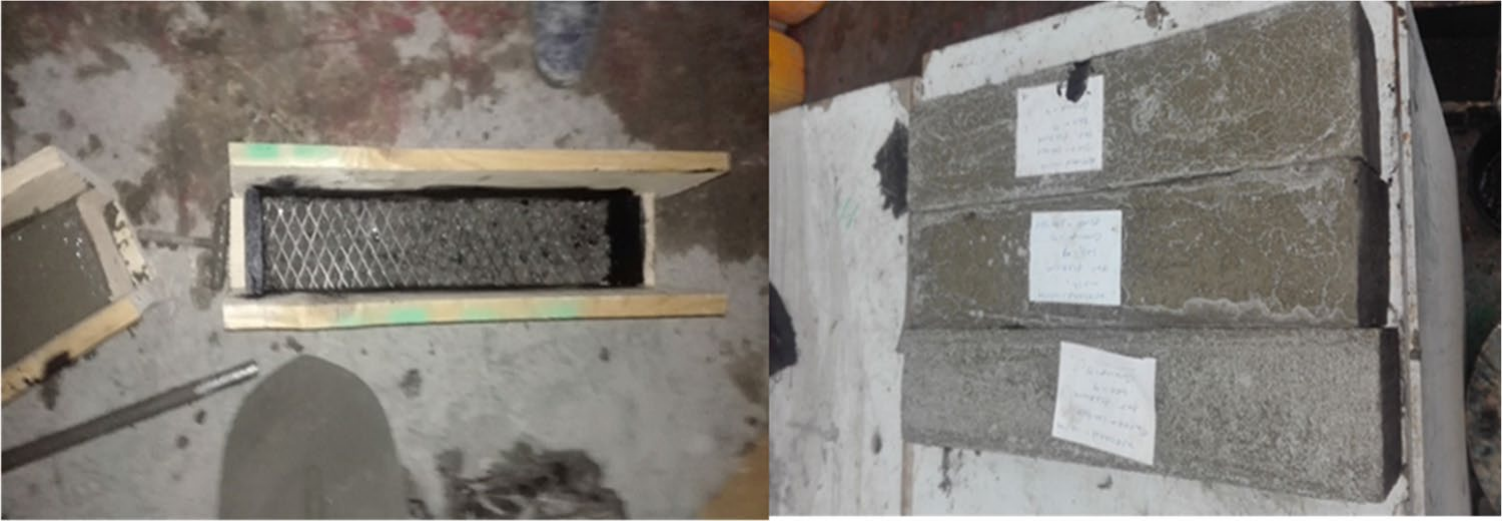
Casting of ferro concrete beam model using expanded metal wire mesh.

Flexural test gives another way of estimation for the tensile strength of concrete. Many heterogeneous aggregate materials, such as rocks, concretes, and certain ceramics, as well as some metals, have improved fracture resistance due to a toughening mechanism caused by the shielding of the crack tip by a nonlinear zone of dispersed microcracking or void formation^48^. The application of ferrocement cover raises the ultimate flexural stress and the first fracture load. The percentage of mesh reinforcement and the thickness of the ferrocement layer increased the first fracture load. For specimens with a ferrocement coating, there was a significant reduction in crack width and spacing (64–84%). In this research, a total of 45 prisms with a total dimension of (100*100*500mm) was casted in a readily made mold. Casting process of the model specimens was done in a special mold prepared from timber due to the absence of enough molds in the laboratory. The flexural test specimens were made up of three different types of gabion reinforcements used in four different set ups. The sample specimens of different wire mesh reinforced concrete models as shown in Figs. 5, 6, and 7.

**Figure 8:**
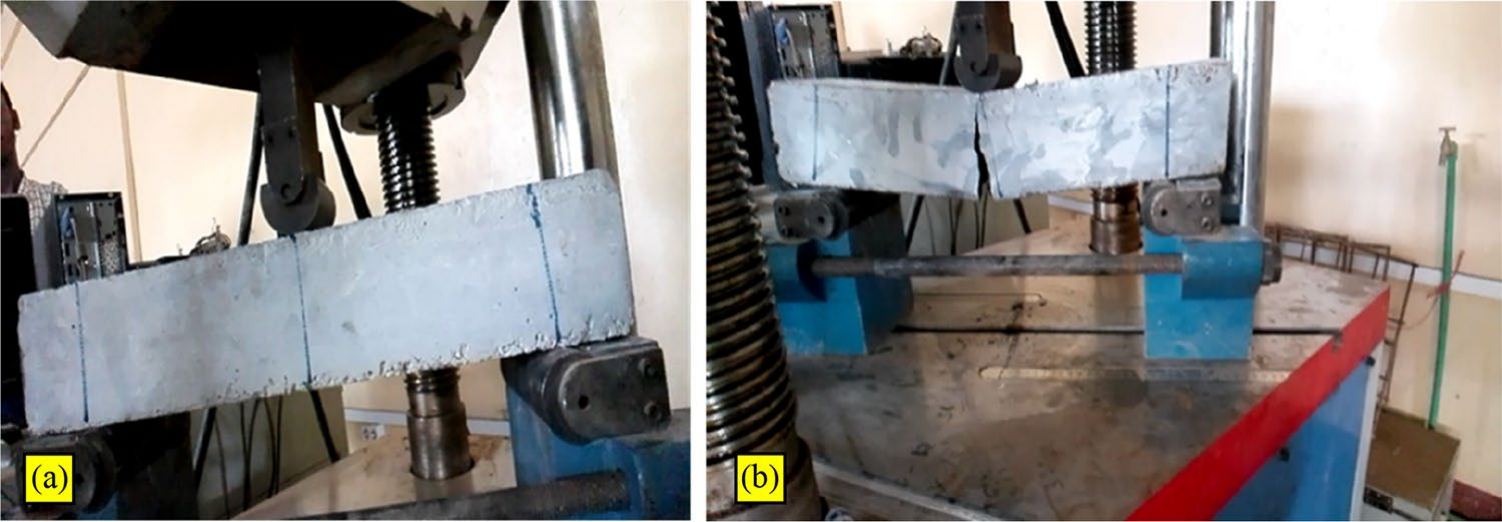
Flexural test (**a**) Specimen during test and (**b**) after ultimate failure.

The testing was made in a universal compression machine with ultimate loading capacity of 1000 kN. The beam was placed between the two jars and load was applied vertically till the cracks appears. Figure 8a depicts the specimen during the test condition while Fig. 8b after the ultimate load when failure occurred.

#### Compressive strength tests

**Figure 9:**
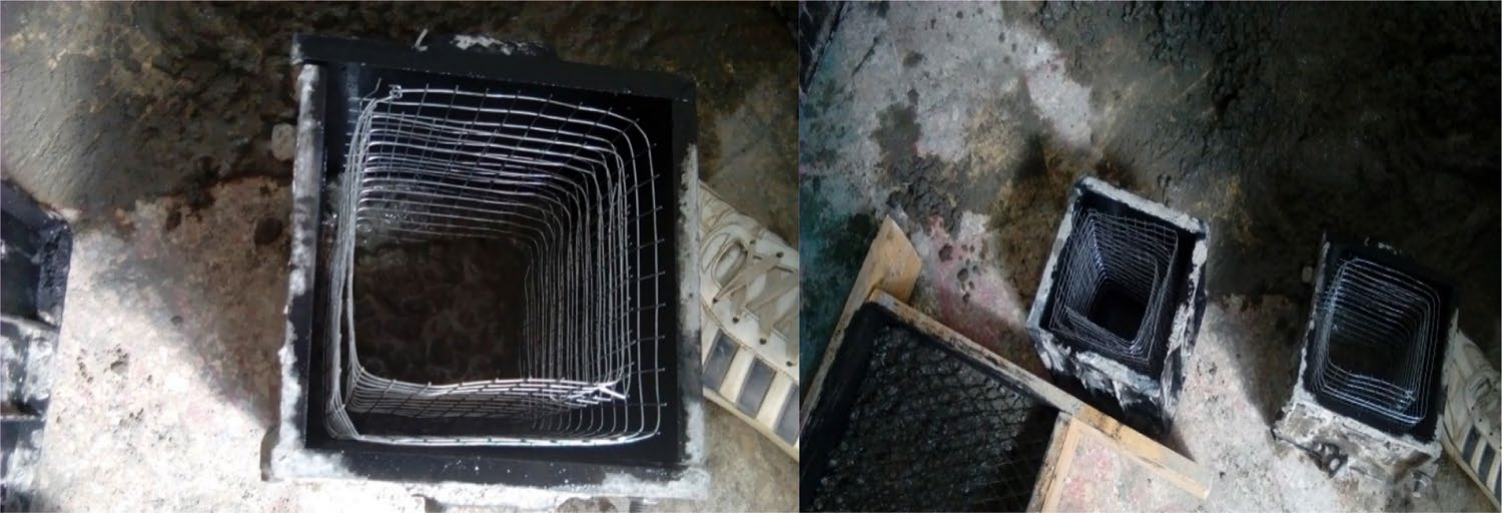
Cubes prepared before concrete casting.

A total of 33 compressive specimens have been prepared for 11 different specimens. A prepared layers of each mesh type according to the number of layers required to the test was prepared and casting of the specimens was made in a standard 150*150*150 cubes readily available in the laboratory. The casting was done by first placing the wire meshes inside the cube by providing the necessary concrete covers as illustrated in the Fig. 9.

**Figure 10:**
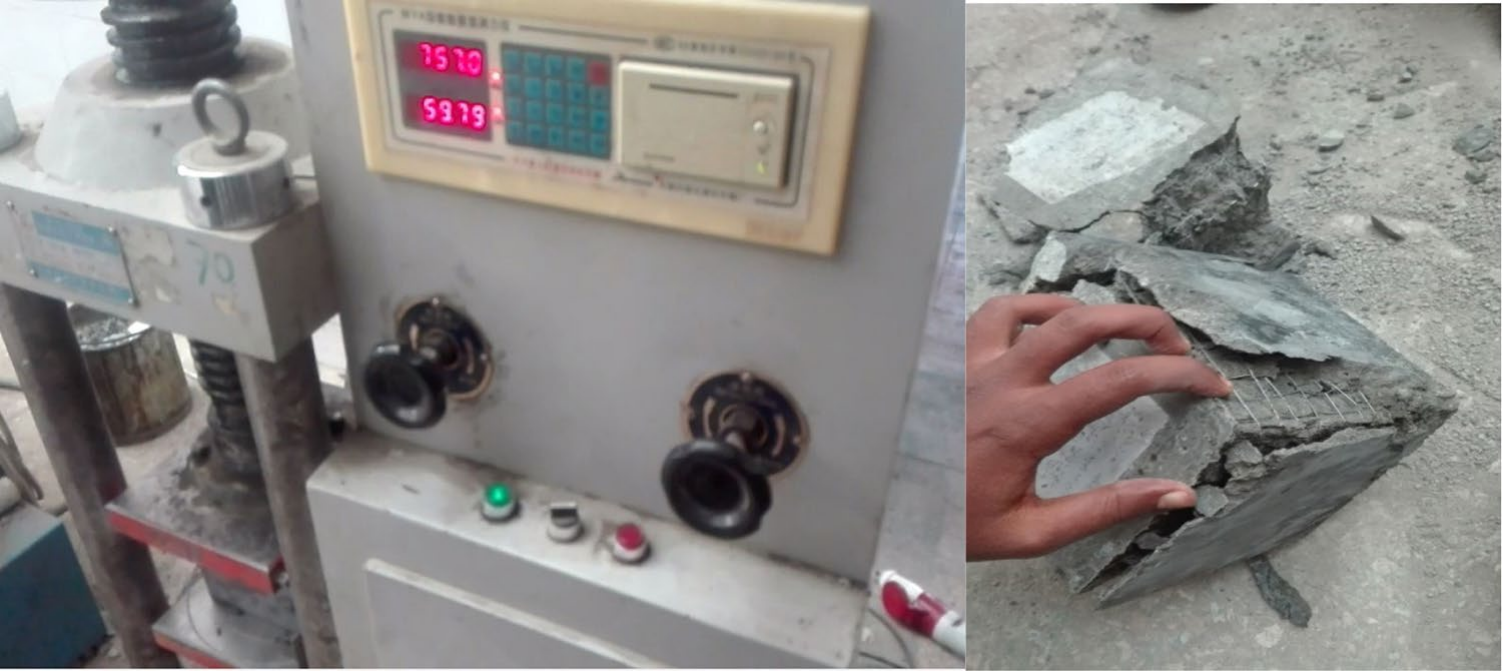
Testing of cube specimen.

The specimens then were allowed to cure for 14 days to attain the maximum strength hence admixture was used. During the specimen preparation due to the interruptions in the power the compaction was made using tamping rod and hand mixes was also used. Finally, the samples were made ready for the testing using universal testing machine. The load was applied vertical at a constant rate till the sample failure as shown in Fig. 10.

#### Energy absorption test

**Figure 11:**
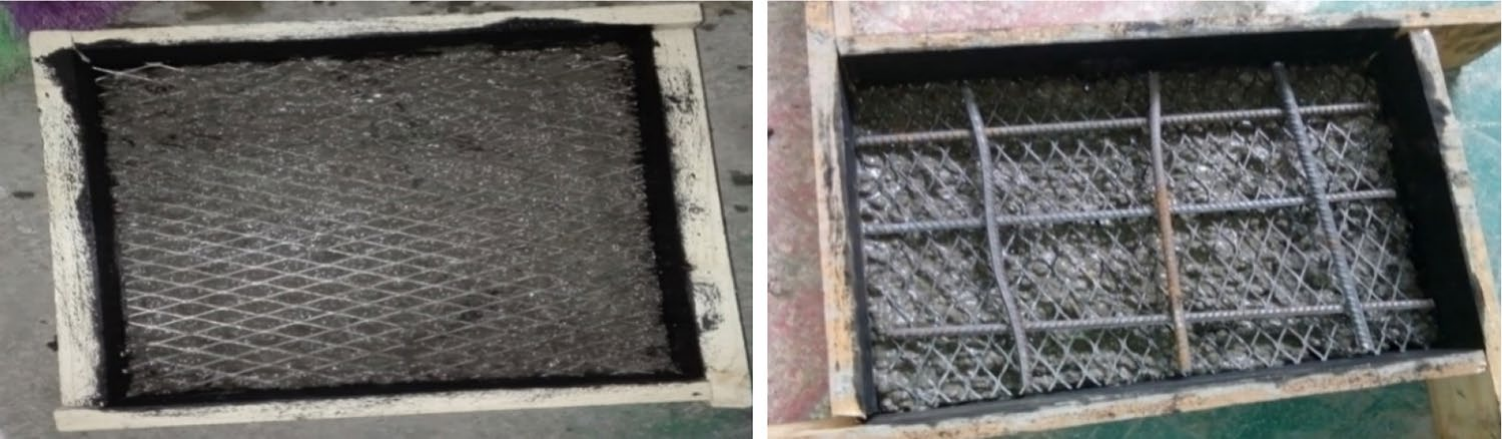
Slab specimens prepared for casting and during casting.

**Figure 12:**
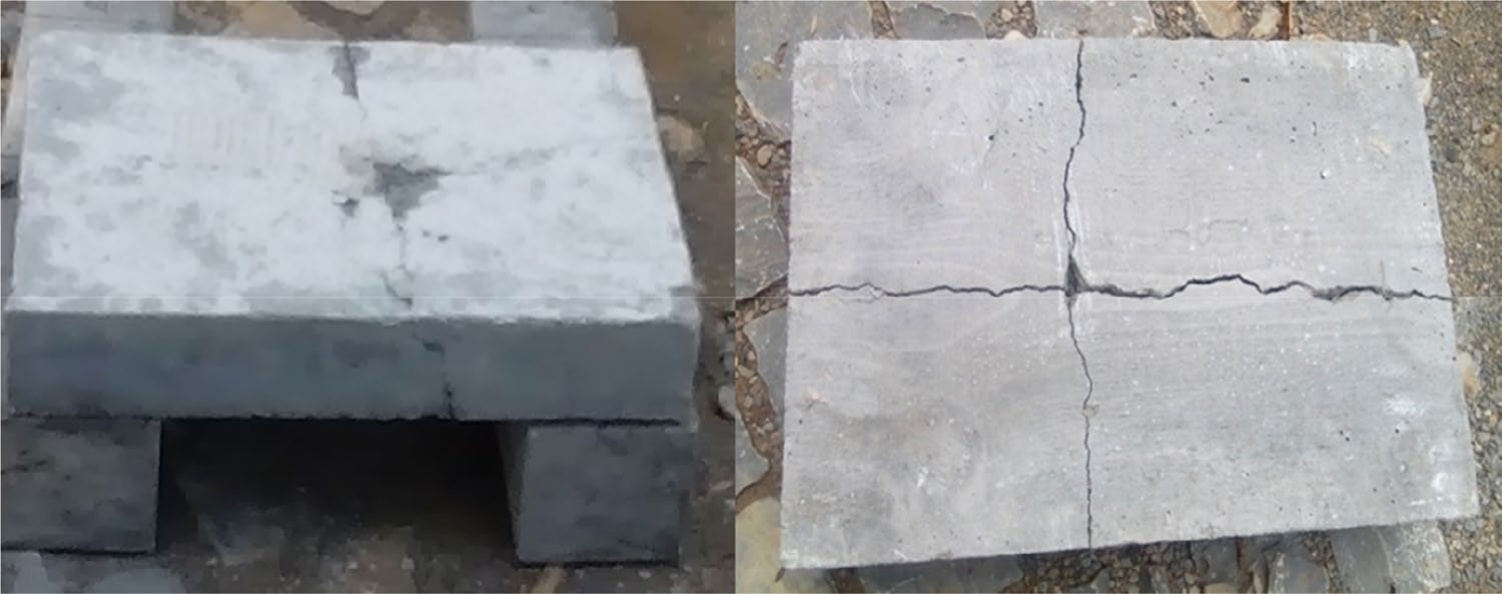
Energy absorption test set up and results after failure.

The crack-arresting mechanism of such composites is improved by the uniform distribution and high surface area-to-volume ratio of the reinforcement (wire mesh)^49^. The wire mesh deformation and failure absorbed more than 80% of the impactor's kinetic energy, while frictional energy dissipation only accounted for roughly 10% of impact energy^50^. A total of 45 specimens have been prepared for energy absorption tests in which the details of the specimen types as shown in Fig. 11. To prepare the different specimens a temporary form work of dimensions 400*300*75 (length, width and thickness) respectively was prepared. Slabs reinforced by different types of mesh and with different number of layers were casted and cured for 14 days. The specimens were prepared as simply supported slab and a 3.028 kg cylindrical steel alloy is allowed to fall freely from a 1.0m height on the top of the slab specimen. The number of blows was recorded at the instant where first crack was observed and at ultimate failure (total collapse of the structure) as shown in Fig. 12.

## Result and discussion

### Flexural strength

Fibers over the neutral axis typically experience CS, and those beneath the neutral axis typically experience tensile stresses, when a bending force is applied downward on a member supported merely at its two ends. Sections of the member close to the supports experience shear stresses that are comparatively larger than tensile stresses in this load and support system. The comparisons of the resistances are presented.

**Figure 13:**
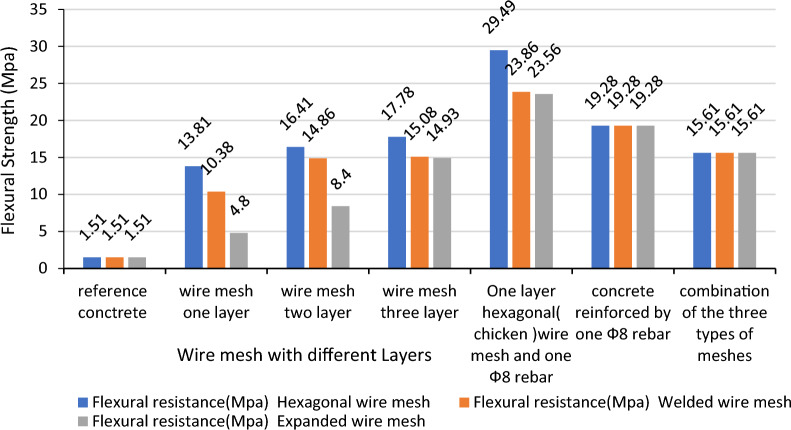
Flexural resistance of reinforced concrete with different wire meshes.

Figure 13 illustrates the laboratory tests of flexural strength of a reinforced concrete beam increases due to addition of one, two and three layers of chicken wire meshes by 814.57%, 986.76% and 1077.48% respectively, welded square wire meshes by 587.42%, 884.11% and 898.68% respectively and expanded metal mesh it is observed to increase by 217.88%, 456.29% and 888.74% respectively while the beam reinforced by the combination of the three has increased the capacity by 933.76%.

The results show significant improvement in the flexural capacity of the beams. On the other hand, though there is an overall increase, the interval of increment doesn’t remain as it is observed in the first layer. The possible reasons for this are:

- The reduction in compaction efficiency as the number of layers increases and
- Inadequate penetration of the aggregate to the openings of the steel wire mesh.

The results show that generally addition of wire meshes to reinforced concrete increases much of the properties of the concrete structures. The flexural strength increases as the number of wire mesh layers increases^51^.

It is observed that as ductility increases, the cracks become increase in number but are very fine textured which are not visible to the casual observer. The increment of the flexural resistance of the reinforced concrete due to the addition of one layer of chicken wire mesh, welded square wire mesh, and expanded metal mesh is 52.96%, 23.76%, and 22.2% respectively. This shows the flexural strength and ductility of the reinforced beam are greater when one layer of chicken wire mesh is added than welded square and expanded metal wire meshes.

The cracks are finer in concrete reinforced by welded square wire mesh than expanded metal and chicken wire meshes in addition to reinforced concrete beams. This is due to the better bonds in the connection of the longitudinal and transversal steel wire meshes in welded square mesh relatively. The chicken wire mesh added reinforced concrete beam exhibits much more deflection than the rest of the other types of wire mesh used for this investigation.

The addition of equivalent two-layer chicken wire mesh increases the flexural capacity by 15.92Mpa, welded square wire mesh by 13.35Mpa, expanded metal mesh by 8.4Mpa, and rebar by 17.71Mpa. As the results show rebar has a better contribution to the flexural resistance of the concrete compared to the equivalent area of the steel wire meshes due to the bond that the material makes with the concrete. The chicken wire mesh performs better than the remaining types of steel wire meshes because of the high yield and ultimate tensile strength compared to the remaining two others. But it is observed that the failure mechanism is ductile, and cracks start at the tension zone and increase upward without visible cracks at the compressive zone even after ultimate failure for all the test specimens. This shows that the failure is tension failure in which the steel yields before the concrete crushes.

In this specific task of the research, it is affirmed once more time again that, the increase in the number of layers increases the flexural capacity of plain concrete (ferro concrete) in line to the works done by researchers in different steel wire meshes for Ferro cement.

To generalize the discussions that are made on the capacities of the different steel wire meshes used as reinforcement for plain concrete and as an additional tension reinforcement, the calculations summarized in Table 8 is used as reference. In general cases, the comparisons made in the three-steel wire meshes are ranked according to the increment due to increasing the number of layers used as reinforcement in the plain concrete. As the calculation results in Table 8 show the average increment in the flexural capacity of the beam is 280.58%, 197.9%, and 123.55% by chicken wire mesh, welded square mesh, and expanded metal mesh layers respectively. According to the summarized results, the overall flexural capacity is increased due to the increase in the addition of chicken wire meshes. This is due to the high tensile and yield strength of the material.

**Table 8:** Average flexural increase due to additional layers of steel wire mesh in %.

Number of layers used in	Flexural resistance due to addition of					
	Chicken wire mesh (Mpa)		Welded square wire mesh (Mpa)		Expanded metal mesh (Mpa)	
	Ultimate	Resistance increment (%)	Ultimate	Resistance increment (%)	Ultimate	Resistance increment (%)
Reference	1.51		1.51		1.51	
One layer	13.81	+ 814.56	10.38	+ 587.41	4.8	+ 217.90
Two layers	16.41	+ 18.83	14.86	+ 4.80	8.4	+ 75
Three layers	17.78	+ 8.35	15.08	+ 1.48	14.93	77.74
Average increment b/n		280.58		197.9		123.55

### Compressive strength

**Figure 14:**
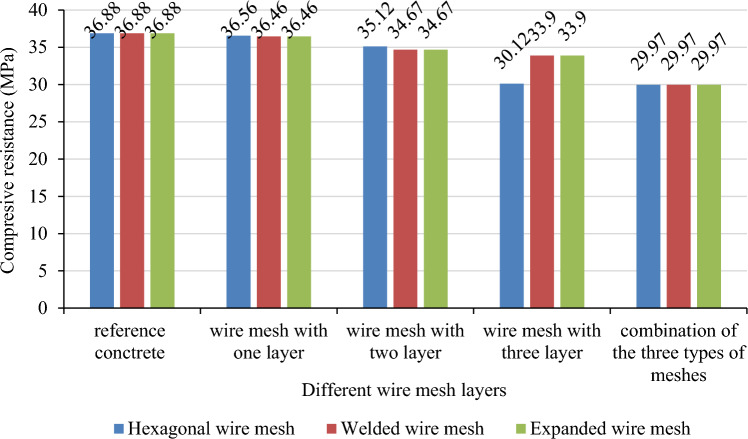
Compressive strength of different metal mesh reinforced concrete.

This section presents the experimental results obtained in various composites. The CS of concrete with LWAC gradually decreases as the number of layers of welded, expanded, hexagonal, and wire mesh increases. The hexagonal wire mesh with a single layer has the greatest CS of 36.56MPa. There is no such variation in the CS of combination of all three types of meshes and were found 29.79MPa. The results from the average axial compressive load testing in chicken wire mesh layered reinforced concrete cube specimens are 36.56Mpa, 35.12Mpa and 30.12Mpa for specimens reinforced by one, two and three layers respectively. The reference concrete on the other hand has highest compressive strength than those results which is 36.88Mpa as shown in Fig. 14. The deviation of the concrete strengths due to the use of one, two and three layers of hexagonal or chicken wire mesh is found − 0.87%, − 4.77% and − 18.33% which is a reduction.

The results from compressive strength tests on welded square wire mesh reinforced concrete cube specimens are 36.46Mpa, 34.67Mpa, 33.9Mpa for one, two and three layers respectively. The deviation in the strength due to the increasing number of welded square wire meshes due to addition of one, two and three layers are − 1.14%, − 5.99% and − 8.08% respectively.

The results from compressive strength tests on expanded metal mesh reinforced concrete cube specimens are 33.66Mpa, 31.74Mpa, and 30.81Mpa for one, two, and three layers respectively. The deviation in the strength due to the increasing number of expanded metal meshes due to the addition of one, two, and three layers are − 8.73%, − 13.94%, and − 16.46% respectively.

Generally, in the entire three tests one similar event was observed, the event of reduction of the compressive strengths in increasing the number of layers used. The results in the above chart show that the addition and increase in the number of layers of the different wire mesh dramatically decrease the compressive strength of the plain concrete. The reasons for this are the presence of structural cracks formed before load is applied because of the weak bond between the wire mesh and adjacent concrete, the high buckling effect of the steel wire meshes, the reduction in the concrete cross-section due to the immersion of smooth textured thin and weak wires and inefficiencies in the compaction of the concrete to create a strong interlocking bond between concrete and adjacent steel wire meshes. Due to the buckling effect and the weak joints in the reinforced concrete specimen failure of the concrete under compressive loading was ductile, rather than brittle type. As all results from the three tests shown in the chart, the responses of all types of steel wire mesh reinforced concrete cube specimens show similar trends of failure.

Taking the average reduction of the compressive strengths due to addition of one more layer presented in the Table 9, it is clear that addition of one layer of hexagonal (chicken) wire mesh to the concrete specimen reduces its compressive strength by 7.66% in average which has the maximum effect in reducing the strength followed by expanded metal mesh(5.79%) and least effect relatively was observed by welded square wire mesh (4.58%) average reduction between consecutive addition of one extra layer to the concrete respectively.

**Table 9:** Comparisons of the three types of meshes performance in compression.

Number of layers	Compressive strength of the cubes due to addition of					
	Chicken wire mesh (Mpa)		Welded square wire mesh (Mpa)		Expanded metal mesh (Mpa)	
	Ultimate resistance	Resistance increments in consecutive layers (%)	Ultimate resistance	Resistance increments in consecutive layers (%)	Ultimate resistance	Resistance increments in consecutive layers (%)
Reference	36.88		36.88		36.88	
One layer	36.56	− 0.87	36.46	− 1.14	33.66	− 8.73
Two layers	35.12	− 7.87	34.67	− 4.91	31.74	− 5.7
Three layers	30.12	− 14.24	33.9	− 7.7	30.81	− 2.93
Average increment		− 7.66%		− 4.58%		− 5.79%

### Energy absorption

In this test the data gathered from the manual experiment was based on the free fall fixed mass spherical steel mass brought in to contact to the slab set up as simply supported specimen which the mass was dropped from a net height of 1.0 m. The number of blows that needed to break or to create cracks in the face of the reinforced concrete slabs was recorded and changed to potential energy using the formula E = mgh, as presented in the Table 3, appendix II of this report. The amount of energy per one blow is calculated as (mgh = 3.028kg * 9.81m/s^2^ * 1.0m = 29.7 J/blow).

**Figure 15:**
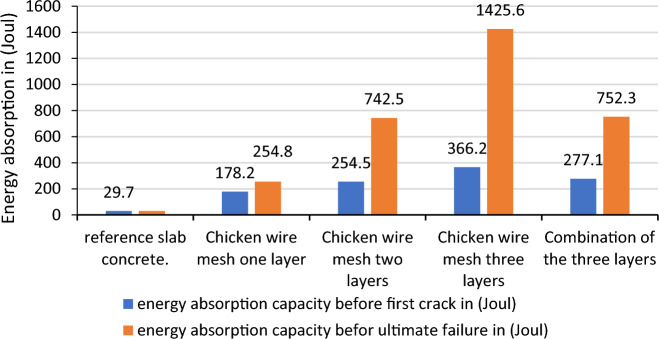
Energy absorption capacity of chicken wire mesh reinforced concrete slab.

**Figure 16:**
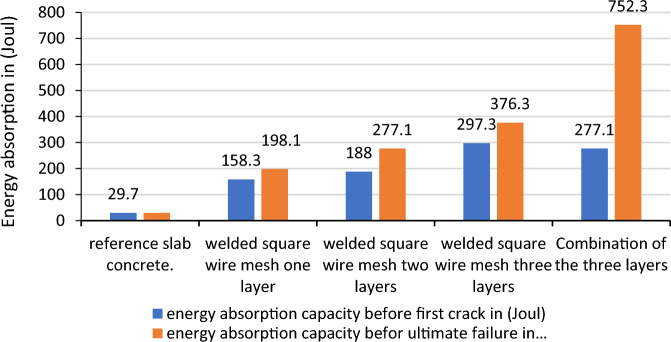
Energy absorption capacity of welded square wire mesh reinforced concrete slab.

**Figure17:**
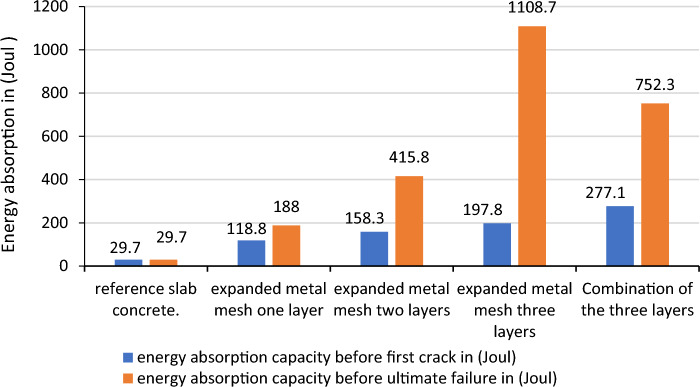
Energy absorption capacity of expanded metal mesh reinforced concrete slab.

The highest energy was recorded in the expanded and chicken wire mesh with three layers before the ultimate failure that 1108.7 and 1425.6 Joul as shown in Figs. 15 and 16 whereas, for welded square wire mesh, the highest energy absorption were found 752.3 Joul in the combination of three layer as shown in Fig. 17. For the first layer of hexagonal wire mesh reinforced concrete, the energy absorption was increased by 82.81% before crack whereas, it increases 88.34% before ultimate failure. The energy absorption capacity of improved ferro cement (ferro concrete) is found 500% and 759.9%, 756.9% and 2400% and 1136% and 4700% respectively for one layer, two layers and three layers of chicken wire mesh reinforced concrete slabs before formation of first crack and ultimate failure is observed.

The energy absorption capacity of improved ferro cement (ferro concrete) is found 433% and 567%, 533% and 833% and 901% and 1167% respectively for one layer, two layers and three layers of welded square wire mesh reinforced concrete slabs before formation of first crack and ultimate failure is observed.

The energy absorption capacity of improved ferro cement (ferro concrete) is found 300% and 533%, 433% and 1300% and 566% and3633% respectively for one layer, two layers and three layers of expanded metal mesh reinforced concrete slabs before the creation of the first crack and before ultimate failure is observed.

The yield and ultimate fracture energy absorption capacities of the specimen made up of the combination of the three different steel wire mesh types were found to increase the resistance by 833% and 2433% in comparison to the energy absorption capacity of reference concrete. While the use of two-way Ф8 reinforcement increases the resistance before the first crack and ultimate failure by 10,400% and 27,360%.

**Figure 18:**
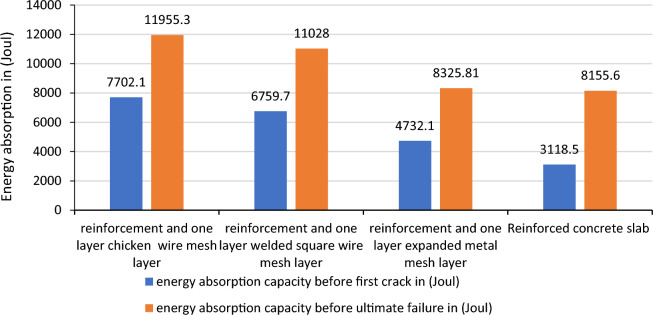
Energy absorption capacity of reinforced concrete additionally reinforced by different steel wire meshes.

The comparisons made on the resistances between reinforced concrete and reinforced concrete additionally reinforced by one layer of the different wire mesh layers showed that considerable improvements are shown in the energy absorption of the slabs as shown in Fig. 18. An addition of one layer of chicken wire mesh welded square wire mesh and expanded metal mesh to the reinforced concrete slab increases the capacity by 146.98% and 46.60%, 116.76% and 35.22%, 51.74%and2.1% before formation of first crack and ultimate failures respectively. As this result shows the expanded metal mesh has the weakest performance compared to the other steel wire meshes. The reasons are due to its in continuity in the connections between the adjacent wires that make up the wire mesh and also the weak orientation of the wire mesh while the slab was casted. It is observed that fine cracks were formed when additional wire meshes were used in the reinforced concrete. The failure was also exhibited by the concrete crush and then steel yield.

To summarize the discussion, the results found in the energy absorption capacity tests of the slabs using the different types of wire meshes shows tremendous improvements in the property of the slab like ductility, toughness and crack restraining behaviours.

So, to come up with the differences in over all performances of the different steel wire meshes the following Tables 10 and 11 are used to conclude the best material in overall results. The criterion used to separate all these materials is the average increment in addition of layers of the steel wire meshes. Accordingly chicken wire mesh with an average increase of the absorption capacity between additional consecutive layers by 195.57% and 347.1% respectively the resistance before the formation of the first crack and ultimate failures ranked first, welded square wire mesh with 169.63% and 214.23% second and expanded metal mesh with 119.3%and 273.6% 5 respectively third. As a result, chicken wire mesh is found best material in overall.

**Table 10:** Increment of energy absorption capacity in addition of consecutive layers before first crack formation.

Number of layers	Energy absorption capacity of the slabs due to addition of					
	Chicken wire mesh		Welded square wire mesh		Expanded metal mesh	
	Resistance before first crack formation	Resistance increments in consecutive layers (%)	Resistance before first crack formation	Resistance increments in consecutive layers (%)	Resistance before first crack formation	Resistance increments in consecutive layers (%)
Reference	29.7		29.7		29.7	
One layer	178.2	500	158.3	433	118.8	299.7
Two layers	254.5	42.82	188	17.76	158.3	33.25
Three layers	366.2	43.89	297.3	58.14	197.8	24.95
Average increment by one layer		195.57		169.63		119.3

**Table 11:** Increment of energy absorption capacity in addition of consecutive layers before ultimate failure formation (%).

Number of layers	Energy absorption capacity of the slabs due to addition of					
	Chicken wire mesh (Joule)		Welded square wire mesh (Joule)		Expanded metal mesh (Joule)	
	Ultimate resistance	Increment of resistance in consecutive layers (%)	Ultimate resistance	Increment of resistance in consecutive layers (%)	Ultimate resistance	Increment of resistance in consecutive layers (%)
Reference	29.7		29.7		29.7	
One layer	254.8	757.9	198.1	567.0	188	533.0
Two layers	742.5	191.41	277.1	39.88	415.8	121.17
Three layers	1425.6	92	376.3	35.8	1108.7	166.64
Average increment by one layer		347.1		214.23		273.6

## Conclusion

The experimental results shown that a concrete reinforced by steel wire meshes generally increases its resistance to flexural applied loadings. The energy absorption capacity increased as the increase in the number of layers. However, the compressive strength of the concrete was found decreased due to the addition and increase in the number of layers of steel wire mesh due to several factors which are interfacial bonding, aggregate segregation, increased Porosity, mesh interaction, reduced cementitious content, cracks and fracture paths. But it was noted that the failure mechanisms in all the specimens tested under the three tests were ductile and the number of cracks increases, and the width of cracks decreases as the number of layers increases especially in the specimens tested for flexure and energy absorption tests. The nature of ductile failure in wire mesh ferrocement contributes to its overall performance by enhancing crack control, energy absorption, durability, structural redundancy, and repairability. These characteristics make wire mesh ferrocement a desirable material for a wide range of construction applications where resilience, ductility, and long-term performance are essential considerations. So, based on the test results the following conclusions have been drawn.

- Wire mesh when used as an additional reinforcement in the beam, enhanced the flexural behavior of the beam by distributing the forces along the section.
- Compared to the control beam, the ultimate moment capacity (the ultimate strength) of the beams has increased by an average of 280.85%, 197.9%, and 123.5% due to the addition of one layer of chicken wire mesh, welded square mesh and expanded metal meshes respectively. Accordingly, the addition of chicken wire mesh performs outperform than the rest of wire meshes.
- The use of wire mesh has made a significant effect on the crack pattern of the reinforced concrete beams, by delaying the crack appearance, increasing the number of cracks, and reducing the crack width. The use of wire mesh reinforcement in concrete beams has a significant effect on crack patterns, structural integrity, and overall performance. By controlling crack formation and propagation, wire mesh reinforcement enhances the strength, durability, and serviceability of concrete beams, ultimately contributing to the safety and reliability of reinforced concrete structures.
- The failure mechanisms of the cube specimens are ductile due to the presence of steel wire meshes.
- The energy absorption capacity of steel wire mesh reinforced concrete specimens is found significantly improved.

Finally, chicken wire mesh is found to be the best steel wire mesh that needs further studies in order to apply in real-life problem-solving projects to construct ferro concrete structures. Future studies need to address the tensile strength, yield strength, and modulus of elasticity with chicken wire mesh. As chicken wire mesh is made of galvanized steel; therefore, its long-term corrosion resistance in a cementitious environment needs to be studied. Research should examine the susceptibility of chicken wire mesh to corrosion and the effectiveness of corrosion protection measures, such as coatings or inhibitors. The optimal configuration of chicken wire mesh in ferrocement structures, including wire diameter, mesh spacing, and mesh orientation should be investigated by future studies. Besides, the structural performance should be investigated in various loading conditions. To sum up, further research is needed to advance the understanding and application of chicken wire mesh in ferrocement structures, addressing key technical, economic, and regulatory considerations to enable its widespread adoption in real-life projects.

## Data Availability

The data used in the present research will be provided upon request.
